# GSE4, a Small Dyskerin- and GSE24.2-Related Peptide, Induces Telomerase Activity, Cell Proliferation and Reduces DNA Damage, Oxidative Stress and Cell Senescence in Dyskerin Mutant Cells

**DOI:** 10.1371/journal.pone.0142980

**Published:** 2015-11-16

**Authors:** Laura Iarriccio, Cristina Manguán-García, Laura Pintado-Berninches, José Miguel Mancheño, Antonio Molina, Rosario Perona, Leandro Sastre

**Affiliations:** 1 Instituto de Investigaciones Biomédicas CSIC/UAM, Madrid, Spain; 2 CIBER de Enfermedades Raras, Valencia, Spain; 3 IdiPaz, Madrid, Spain; 4 Instituto de Química-Física “Rocasolano” CSIC, Madrid, Spain; 5 Advanced Medical Projects, Madrid, Spain; 6 Biomarkers and Experimental Therapeutics in Cancer, IdiPaz, Hospital Universitario La Paz, Madrid, Spain; Tulane University Health Sciences Center, UNITED STATES

## Abstract

Dyskeratosis congenita is an inherited disease caused by mutations in genes coding for telomeric components. It was previously reported that expression of a dyskerin-derived peptide, GSE24.2, increases telomerase activity, regulates gene expression and decreases DNA damage and oxidative stress in dyskeratosis congenita patient cells. The biological activity of short peptides derived from GSE24.2 was tested and one of them, GSE4, that probed to be active, was further characterized in this article. Expression of this eleven amino acids long peptide increased telomerase activity and reduced DNA damage, oxidative stress and cell senescence in dyskerin-mutated cells. GSE4 expression also activated c-myc and TERT promoters and increase of c-myc, TERT and TERC expression. The level of biological activity of GSE4 was similar to that obtained by GSE24.2 expression. Incorporation of a dyskerin nuclear localization signal to GSE24.2 did not change its activity on promoter regulation and DNA damage protection. However, incorporation of a signal that increases the rate of nucleolar localization impaired GSE24.2 activity. Incorporation of the dyskerin nuclear localization signal to GSE4 did not alter its biological activity. Mutation of the Aspartic Acid residue that is conserved in the pseudouridine synthase domain present in GSE4 did not impair its activity, except for the repression of c-myc promoter activity and the decrease of c-myc, TERT and TERC gene expression in dyskerin-mutated cells. These results indicated that GSE4 could be of great therapeutic interest for treatment of dyskeratosis congenita patients.

## Introduction

Telomere maintenance alterations are in the origin of an increasing number of diseases such as dyskeratosis congenita, aplastic anemia or pulmonary fibrosis (recently reviewed by S.A. Savage [1]). Telomeres are structures located at the end of the chromosomes that play essential roles in chromosome replication and stability [2, 3]. The sequence of their DNA consists of hundreds of repeats of the TTAGGG motif. The DNA replication machinery cannot complete the synthesis of the chromosome ends that is accomplished by a RNA-protein complex with reverse transcriptase activity named telomerase [4]. The telomerase protein with reverse transcriptase activity is encoded by the TERT gene and uses as template the RNA molecule encoded by the TERC (also named TR) gene that is another component of the telomerase complex [5]. A third essential component is dyskerin, encoded by the dkc1 gene [6, 7]. Additional components of the telomerase complex include the proteins NOP10, GAR and NHP2 [8]. Telomeres acquire a very specialized structure since the terminal region of the DNA stays single-stranded and folds back to get inter winged with a close telomere region to form a circular structure (T-circle) [9]. In addition, the telomere DNA binds to a specific protein complex, named shelterin complex, which protects telomeres from degradation [10]. This structure also avoids the recognition of telomeres as damaged DNA by the DNA-repair signalling system. The correct structure of the telomeres is therefore essential for the maintenance of chromosome integrity and cell cycle progression [11]. Telomere shortening that occurs during proliferation of non-stem or transformed cells results in genome instability, the fusion of chromosomes and induces apoptotic cell death or senescence [11].

Mutations in the genes coding for components of the telomerase (TERT, TERC, DKC, NOP10, NH2) or shelterin (TINF2) complexes cause a number of diseases known as telomeropathies or Telomere Biology Disorders. Among them are dyskeratosis congenita, premature aging syndromes, aplastic anemia, pulmonary fibrosis and cancer (see Savage, S.A. [1] and Glousker, G. et al [12] for recent reviews).

Dyskeratosis congenita is a rare disorder characterized by bone marrow failure and increased susceptibility to cancer [13]. Mutations in DKC1 produce the predominant X-linked form of this disease. The encoded protein, dyskerin, is a pseudouridine synthase required for the postranscriptional modification of ribosomal, small nuclear and nucleolar RNAs and some mRNAs [7, 14] [15, 16]. In addition, is an essential component of the telomerase complex as previously indicated. Dyskerin has three conserved domains, the Dyskerin Like Domain (DKLD), the pseudouridine synthase domain (TRUB domain) and the RNA binding domain (PUA domain) [7]. Mutations in these domains produce X-linked dyskeratosis congenita [7, 17]. We have previously described that a 55 amino acids-long fragment of the dyskerin TRUB domain, named GSE24.2, has protective effects on cells derived from dyskeratosis congenita patients [18]. GSE24.2 treatment increases telomerase activity of patient cells. This peptide also protects cells from treatment with the anticancer drug cisplatin, that induces intra- and inter-strand DNA bridges, and from telomerase inhibitors. Expression of GSE24.2 from plasmid or viral vectors or direct transfection of cells with the peptide, produced in bacteria or chemically synthesized, have similar effects [19]. GSE24.2 increases TERT and c-myc expression through transcriptional activation and stabilizes TERC RNA in dyskerin mutant cells [19]. This peptide protects cells from basal DNA damage, which is increased in dyskeratosis congenita patients [20]. These activities make of GSE24-2 a good candidate for a therapeutic approach to dyskeratosis congenita and related telomeropathies. Actually, the EMA recently approved GSE24.2 as an orphan drug for dyskeratosis congenita treatment (EU/3/12/1070-EMA/OD/136/11).

In this article we describe that a smaller peptide of just eleven amino acids, named GSE4, corresponding to the N-terminal region of GSE24.2, maintains the same capacity to regulate gene expression, to protect cells from DNA damage and to decrease oxidative stress as GSE24.2. In addition, GSE24.2 and GSE4 protect dyskeratosis congenita patient’s cell from cell senescence.

## Materials and Methods

### Cell culture and transfection

The previously described F9 and dyskerin-mutated F9 cells (F9-A353V) [19, 21], were cultured in Dulbecco Modified Eagle Medium (DMEM) supplemented with 10% foetal bovine serum, 2 mM glutamine (Gibco) and 1.5 gr/ml of sodium bicarbonate. HEK293T cells (ATCC) were cultured in DMEM media supplemented with 10% foetal bovine serum and 2 mM glutamine. DC-C and DC-3 cells (Coriell Cell Repository) were cultured in Roswell Park Memorial Institute (RPMI) media supplemented with 20% foetal bovine serum and 2 mM glutamine. XDC-1787-C (Corriel Cell Repository) and F26IIB cells (generated in our laboratory), were cultured in Minimum Essential Medium Eagle (MEM) media supplemented with 15% foetal bovine serum and 2 mM glutamine. Cells were transfected with 10 μg of DNA/10^6^ cells using lipofectamine plus (Invitrogene, Carlsbad, USA) or the K2 transfection kit (Biontex Lab, Planegg/Martinsried, GE) according to the manufacturers’ instructions. 15 μg of peptides per 35 mm dish were transfected using the ProteoJuice Protein Transfection Reagent (Merck Millipore, Billerica, MA, USA) or the K2 transfection kit.

### Peptide production and purification

The GSE24.2 peptide was produced in E. coli DH5α transformed with pGATEV GSE24.2 as previously described [20]. HPLC-purified synthetic peptides were obtained from Peptide 2.0 Inc (Chantilly, USA) and China Peptides Co. Ltd. (Shanghai, China).

### Vector constructions and in vitro mutagenesis

The localization signal KRKR (amino acids 446–449) was incorporated to the 3’ end of the GSE 24.2 expression vector by PCR using the oligonucleotides 5’-GGGAATTCTGGTTTGATTAATCTTGACAAGC-3’ and 5’-GGTCTAGACTCGCTTCCGCTTCTTCACCAAGCGAGTGGCTCG-3’. The amplified product was cloned between the EcoRI and XbaI sites of the pcDNA3-myc vector to generate the GSE24.2-NLS1.3 vector. The localization signal KKEKKKSKK (amino acids 467–476) was introduced using the oligonucleotides: 5’-GGGAATTCTGGTTTGATTAATCTTGACAAGC-3’ and 5’-GGTCTAGACCTTCTTACTCTTCTTCTTTTCCTTCTTCTTCACCAAGCGAGTGGCTCG-3’ and cloned in pcDNA3-myc to generate GSE24.2-NLS2.3. The fragments GSE24.2, GSE24.2-NLS1.3 and GSE24.2-NLS2.3 were also cloned in the pRRL-CMV-IRES-GFP vector using the PstI and BamHI restriction sites. GSE4 and derived fragment were cloned between the PstI and BamHI restriction sites of pRRL-CMV as double-stranded oligonucleotides obtained by hybridization of the following oligonucleotides: GSE4, 5’-GATGGGTTTCATTAATCTTGACAAGCCCTCTAACCCCTAAG-3’ and 5’-GATCCTTAGGGGTTAGAGGGCTTGTCAAGATTAATGAAACCCATCTGCA-3’; GSE4-NLS1: 5’-GATGGGTTTCATTAATCTTGACAAGCCCTCTAACCCCAAGCGGAAGCGATAAG-3’ and 5’-GATCCTTATCGCTTCCGCTTGGGGTTAGAGGGCTTGTCAAGATTAATGAAACCCATCTGCA-3’; GSE4-NLS1-DA (incorporating the GAC to GCC change): 5’-GATGGGTTTCATTAATCTTGCCAAGCCCTCTAACCCCAAGCGGAAGCGATAAG-3’ and 5’-GATCCTTATCGCTTCCGCTTGGGGTTAGAGGGCTTGGCAAGATTAATGAAACCCATCTGCA-3’.

### Gene-reporter assays

Cells were transfected with 10 μg/10^6^ cells of expression vectors for GSE24.2, GSE4 and derived peptides and the corresponding luciferase reporter vectors (1 μg/10^6^ cells). The TERT and c-myc (px3.2) promoter constructs have been described previously [18, 22]. Twenty four hours after transfection protein extracts were prepared and luciferase activity determined using a commercial kit (Promega Corporation, Madison, WI, USA). Luciferase activity was expressed as arbitrary units by μg of protein concentration, determined with the Bradford Reagent (BioRad, Berkeley, CA, USA).

### Immunofluorescence and immunocytochemistry

Cells were grown on coverslips, fixed, permeabilized and incubated with the corresponding antibodies as previously described [20]. Nucleoli were stained incubating the cells for 20 min with 0,5 μM of the SYTO RNA Select Green Fluorescent Cell Stain (Molecular Probes). DNA damage foci were identified by the presence of γH2AX as previously described [20]. Oxidative stress was determined using the anti-8-oxoguanine antibody, clone 483.15 (MAB3560, Merck-Millipore). Images were acquired using a Zeiss Confocal microscope and processed using ZEN 2011 Light Edition and ImageJ software. DNA synthesis was determined by immunocytochemistry using anti-Ki67 antibodies (RM9106-50; NeoMarkers Inc. Fremont, CA, USA).

### Telomeric Repeat Amplification Protocol (TRAP) assay

The TRAPeze kit (Millipore, Billerica, MA, USA) [23] was used to determine telomerase activity. The activity of each sample was normalized using the internal control provided in the kit.

### Determination of reactive oxygen species (ROS) content with dihydroethidium

Cells were washed 2 times with pre-warmed PBS medium, dihydroethidium (Dihydroethidium, D7008-Sigma, St. Louis, MI. USA) was added and the cells incubated at 37°C for 25 min. The fluorescence was measured using FACS SCAN II (BD, USA), with 530 nm of excitation wavelength and 630 nm of emission wavelength.

### Determination of cell senescence

Cells were plated on 6-well plates at a density of 1x10^4^ cells/well. After 4 days of culture cells were fixed and the acid-β-galactosidase activity detected using the Senescence Detection Kit (BioVision, USA). Six images were taken from each well using a Nikon Eclipse TS100 microscope (Nikon, USA) and the percentage of senescent cells calculated for each of them.

### Protein expression analyses

Whole-cell extracts were obtained and analyzed by Western blot as previously described [24].

### Antibodies

Primary antibodies used for γH2AX and phosporylated Chk2 detection were from Cell Signalling Technology (2577, Cell Signalling Technology, Danvers, MA, USA), and those used for α-tubulin detection from Sigma-Aldrich (T9026, Sigma-Aldrich, St. Louis, MO, USA). Secondary antibodies were purchased from BioRad (Berkeley, CA, USA) and Cell Signalling Technology.

### Quantitative RT-PCR analyses of gene expression

Total RNA was obtained from the cell cultures using the Trizol Reagent (Invitrogen, Carlsbad, CA, USA). One microgram of RNA was converted to cDNA using random primers and the High-Capacity cDNA Archive Kit (Applied Biosystems, Foster City, CA, USA). Quantitative PCR was done using the Power SYBR Green kit (Applied Biosystems, Foster City, CA, USA). The programs used have been described by Machado-Pinilla, et al. [19]. SOD1(Cu/Zn SOD) and SOD2 (Mn SOD) mRNA levels were determined using the TaqMan Universal PCR Master Mix. The StepOne Plus Real Time PCR System (Applied Biosystems, Foster City, CA, USA) was used for analyses of the PCR products. Relative gene expression was calculated according to the comparative threshold cycle method [25] using β-actin as endogenous control.

### Primers used for quantitative RT-PCR

The following primers were used: m-cmyc-S: 5’-GAGCTGTTTGAAGGCTGGATTT-3’; m-cmyc-AS: 5’-TCCTGTTGGTGAAGTTCACGTT-3’; m-TERT-S: 5’-AGATCAAGAGCAGTAGTCGCCAG-3’; m-TERT-AS: 5’-TTTACAGCACACCGACCCAGAG-3’; m-TR-S: 5’: GCTGTGGGTTCTGGTCTTTTGTTC-3’; m-TR-AS: 5’-CGTTTGTTTTTGAGGCTCGGG-3’; β-actin-S: 5’- GGTATGGAATCCTGTGGCATCCATGAAA-3’; β-actin-AS: 5’-GTGTAAAACGCAGCTCAGTAACAGTCCG-3’.

### Statistical analyses

Experiments were repeated, at least, three times with triplicate samples. Statistical significance was calculated using the Unpaired t-test (two- tailed). The significance has been considered at *p<0.05, **p<0.01 and ***p<0.001. GraphPad software v5.0 was used for statistical analysis and graphic representations.

## Results

### 1. Functional study of the GSE24.2-derived peptide GSE4 on DNA damage rescue, telomerase activity and cell proliferation in dyskeratosis congenita cells

**Fig 1:**
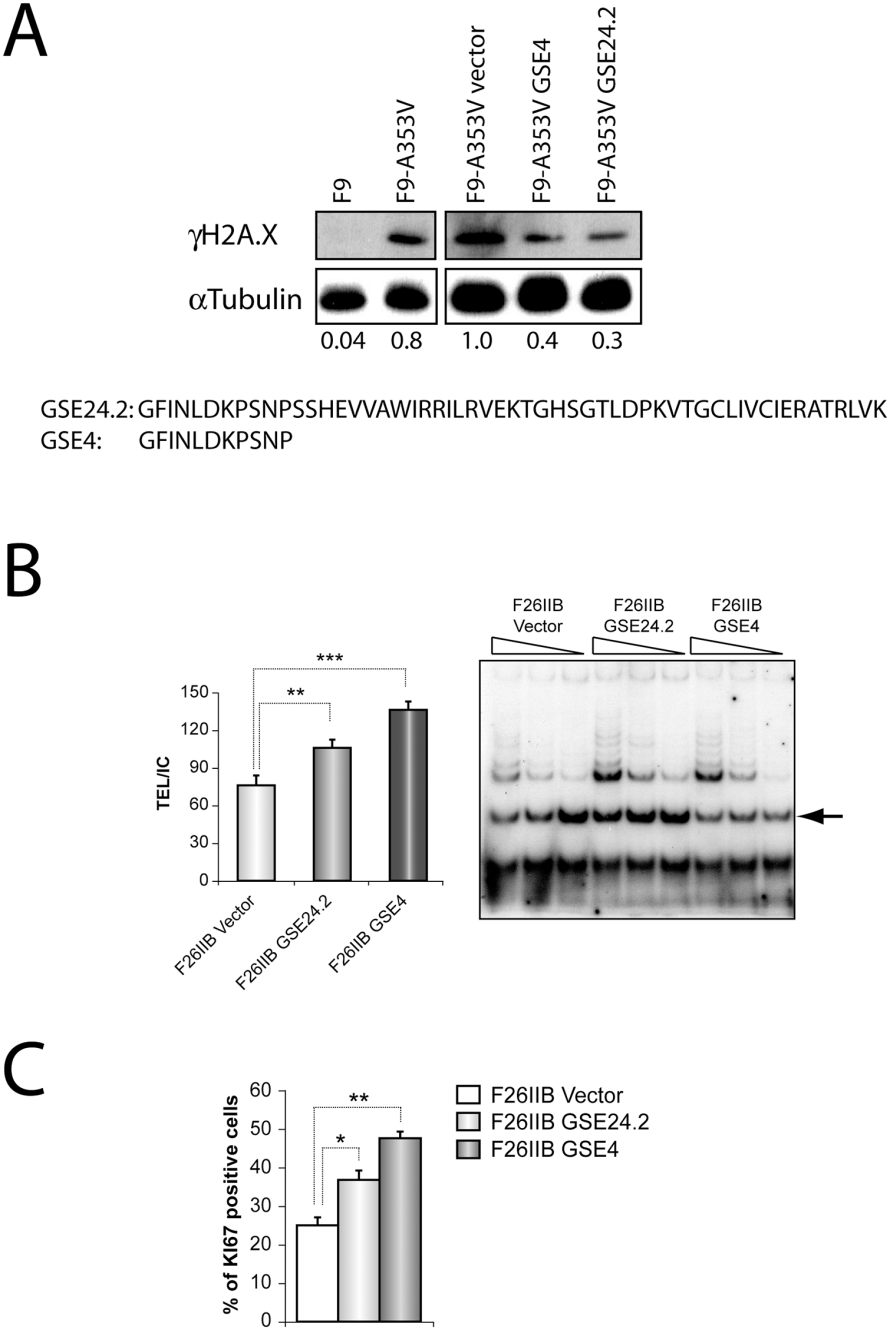
DNA-damage protective effect, telomerase activation and cell proliferation induction of one small peptide, GSE4, derived from GSE24.2. Panel A. One small peptide derived from GSE24.2, GSE4, and GSE24.2 were expressed in F9_A353V cells that were transfected with the pRRL-CMV-IRES-EGFP vector, either empty (Vector) or expressing GSE24.2 or GSE4. Twenty four hours later cells were lysed and the presence of γH2AX and α-tubulin (loading control) analyzed by western blot. Un-transfected F9 and F9-A353V cells were used as controls. The values at the bottom of the panel indicate the estimated ratio between γH2AX and α-tubulin expression levels referred to those found in cells transfected with the empty vector (F9-A353V vector). The amino acid sequences of GSE24.2 and GSE4 are indicated at the lower part of the panel. Panel B. The telomerase activity of F26IIB cells transfected with the pRRL-CMV-IRES-GFP vector empty (vector), expressing GSE24.2 (GSE24.2) or GSE4 (GSE4) was determined using the Telomeric Repeat Amplification Protocol (TRAP) assay. The amplification products obtained using three decreasing amounts of cell extracts for each cell line are shown in the right panel. Quantification of the amplification products, normalized to the internal control provided in the assay (indicated by an arrow at the right panel) is shown in the left panel. Panel C. Expression of Ki67 was determined by immunocytochemistry in F26IIB cells transfected as described in panel B. The percentage of cells expressing Ki67 is represented for each type of transfected cells. The experiments were repeated three times with similar results. Asterisks indicated the statistical significance (* p<0.05, **p<0.01, ***p<0.001).

Many of the previous functional studies were done with GSE24.2 peptides produced in E. coli. The analysis of these preparations by mass spectroscopy indicated the presence of several smaller peptides probably originated by GSE24.2 partial degradation. The more abundant peptide was an eleven amino acids long peptide corresponding to the N-terminal region of GSE24.2 (GSE4, Fig 1A). The functional activity of this peptide was compared to that of GSE24.2 in several analyses. We have previously reported that the GSE24.2 peptide reduced DNA damage [20]. We transfected F9 or the F9-derived dyskerin-mutant F9A353V cells with pRRL-CMV-IRES-GFP vector, either empty (vector) or expressing GSE24.2 or GSE4 to study their possible effect on basal DNA damage by measuring γH2AX phosphorylation (Fig 1A). We found that the amount of **γ**H2AX in F9A353V cells decreased to similar levels after transfecting both peptides (40 and 30%, respectively). The possible effect of GSE4 expression on telomerase activity was assayed on the fibroblastoid cell line F26IIB, derived from a dyskeratosis congenita patient carrying the Ala2Val hemizygous mutation [26]. Cells were transfected with the above-mentioned vectors and their telomerase activity determined, as shown in Fig 1B. Quantification of the extension products indicated a significant increase in telomerase activity upon GSE24.2 and GSE4 expression. The proliferation capacity of the transfected cells was estimated by the expression of the Ki67 nucleolar protein, as shown in Fig 1C. A significantly higher percentage of cells expressing GSE24.2 and GSE4 were positive for Ki67 expression, indicative of higher proliferation rates.

### 2. Oxidative stress and senescence are decreased in X-DC cells by GSE4 expression

**Fig 2:**
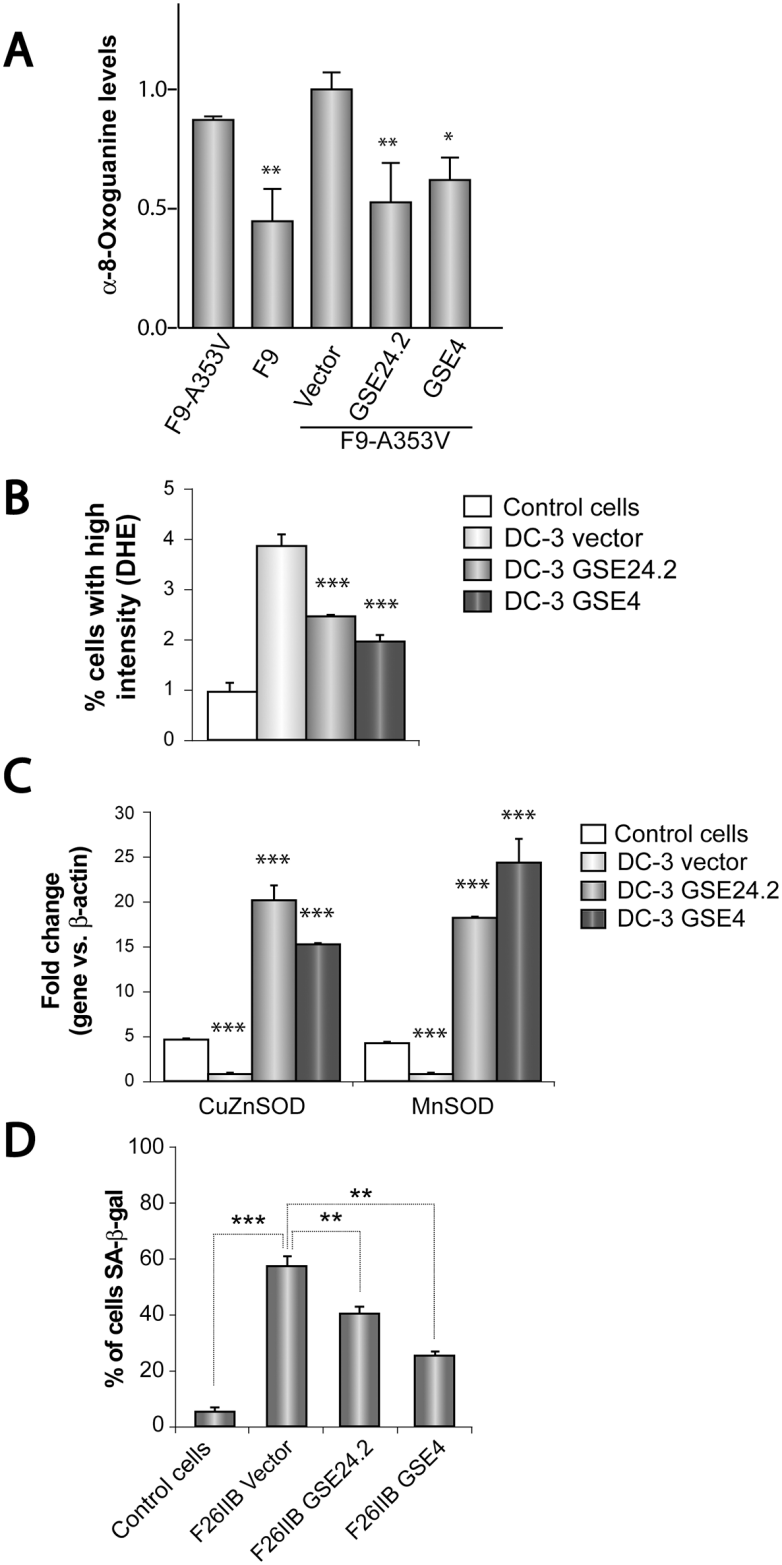
Effects of GSE24 and GSE4 peptides on oxidative stress and cell senescence. Panel A. F9-A353V cells were transfected with the pRRL-CMV-IRES-EGFP vector empty (Vector), expressing GSE24.2 (GSE24.2) or GSE4 (GSE4) (10 μg DNA per million cells). After 24 hours of transfection cells were fixed and incubated with an anti-α-8-Oxoguanine antibody. The amount of antibody bound was determined using a Zeiss Confocal microscope and quantified with the ImageJ program. Untransfected F9 and F9-A353V cells were used as controls. Average α-8-Oxoguanine expression and standard deviations are shown. Panel B. The amount of Reactive Oxygen Species (ROS) of control cells derived from healthy relative controls (DC-C) and cells derived form a dyskeratosis congenita patient (DC-3) infected with the pRRL-CMV-IRES-EGFP vector, either empty (DC-3 vector) or expressing GSE24.2 (DC-3 GSE24.2) or GSE4 (DC-3 GSE4) was determined using dihydroethidium (DHE). The average percentage of cells with high intensity of DHE and standard deviation are represented. Panel C. Cells obtained from dyskeratosis congenita patients and healthy relatives were transfected as described in panel B. The expression of the mRNAs coding for Cu/Zn Superoxide dismutase (CuZnSOD) and Mn Superoxide dismutase (MnSOD) were determined by quantitative RT-PCR. Panel D. The presence of senescent cells in the cell cultures was determined by the assay of the β-Galactosidase activity using the X-Gal substrate. Fibroblasts obtained from a dyskeratosis congenita patient (F26IIB) were transfected as described above using the empty vector (F26IIB vector) or those expressing GSE24.2 (F26IIB GSE24.2) or GSE4 (F26IIB GSE4). Control cells isolated from a healthy relative (XDC-1787C) were used as control. The percentage of cells expressing β-Galactosidase activity (SA-β-gal) is represented for each cell line. Average values and standard deviations obtained from three different experiments are represented. Statistical significance: * p<0.05, **p<0.01, ***p<0.001.

Oxidative stress is one of the causes of DNA damage producing both single-strand breaks (SSBs) and double-strand breaks (DSBs). Cells obtained from dyskeratosis congenita patients showed high levels of oxidative stress and reactive oxygen species (ROS) production that decreased upon treatment with GSE24.2 [20]. We therefore tested the activity of GSE4 on oxidative stress on a cellular model of dyskeratosis congenita. Oxidative stress was first determined by the presence of α-8-oxoguanine on the cellular DNA. F9-A353V cells were transfected with the pRRL-CMV-IRES-GFP vector expressing GSE24.2, GSE4 or the empty vector and the amount of α-8-oxoguanine present on the cells determined by immunohistochemistry. F9 cells that do not carry any DKC mutation were used as control. The quantification of α-8-oxoguanine expression is shown in Fig 2A. Expression of GSE24.2 and GSE4 decreased α-8-oxoguanine levels of F9-A353V cells. Oxidative stress was also measured in cells derived from dyskeratosis congenita patients (DC-3, carrying the Thr66Ala mutation in dkc1) and healthy relatives (DC-C) by determining ROS levels using dihydroethidium, as shown in Fig 2B. Dyskeratosis- congenita patient cells infected with the empty vector showed higher levels of ROS than those of the healthy controls. Expression of GSE24.2 and GSE4 in the patient cells significantly decreased ROS levels. ROS production is associated with decreased expression of antioxidant enzymes such as the Cu/Zn- and Mn-dependent Superoxide Dismutases (SOD) [20]. The expression levels of the mRNAs coding for these two enzymes was determined by RT-qPCR, as shown in Fig 2C. Patient cells expressing the empty vector (DC-3 vector) showed decreased levels of both mRNAs, as compared to cells from the healthy control (Control cells). Expression of GSE24-2 and GSE4 greatly increased Cu/Zn SOD and Mn SOD mRNA expression levels in agreement with their capacity to decrease ROS production.

Telomere shortening has been described to result in cell senescence [27]. Therefore, the possible effect of GSE24.2 and GSE4 expression in senescence of dyskeratosis congenita patient’s cells has been studied. One fibroblastoid cell line established from a dyskeratosis congenita patient (F26IIB) was transfected with the pRRL-CMV-IRES-GFP vector, either empty or expressing GSE24.2 or GSE4. After four days of culture senescent cells were identified by the expression of acidic β-galactosidase and the percentage of senescent (SA-β-gal) cells determined, as shown in Fig 2D. A fibroblastoid cell line established from a healthy relative of a DC patient (XDC-1787-C) was used as internal control. Expression of GSE4 and GSE24.2 significantly decreased cell senescence.

### 3. Requirement of nuclear and nucleolar localization signals for GSE24.2 and GSE4 biological activity

**Fig 3:**
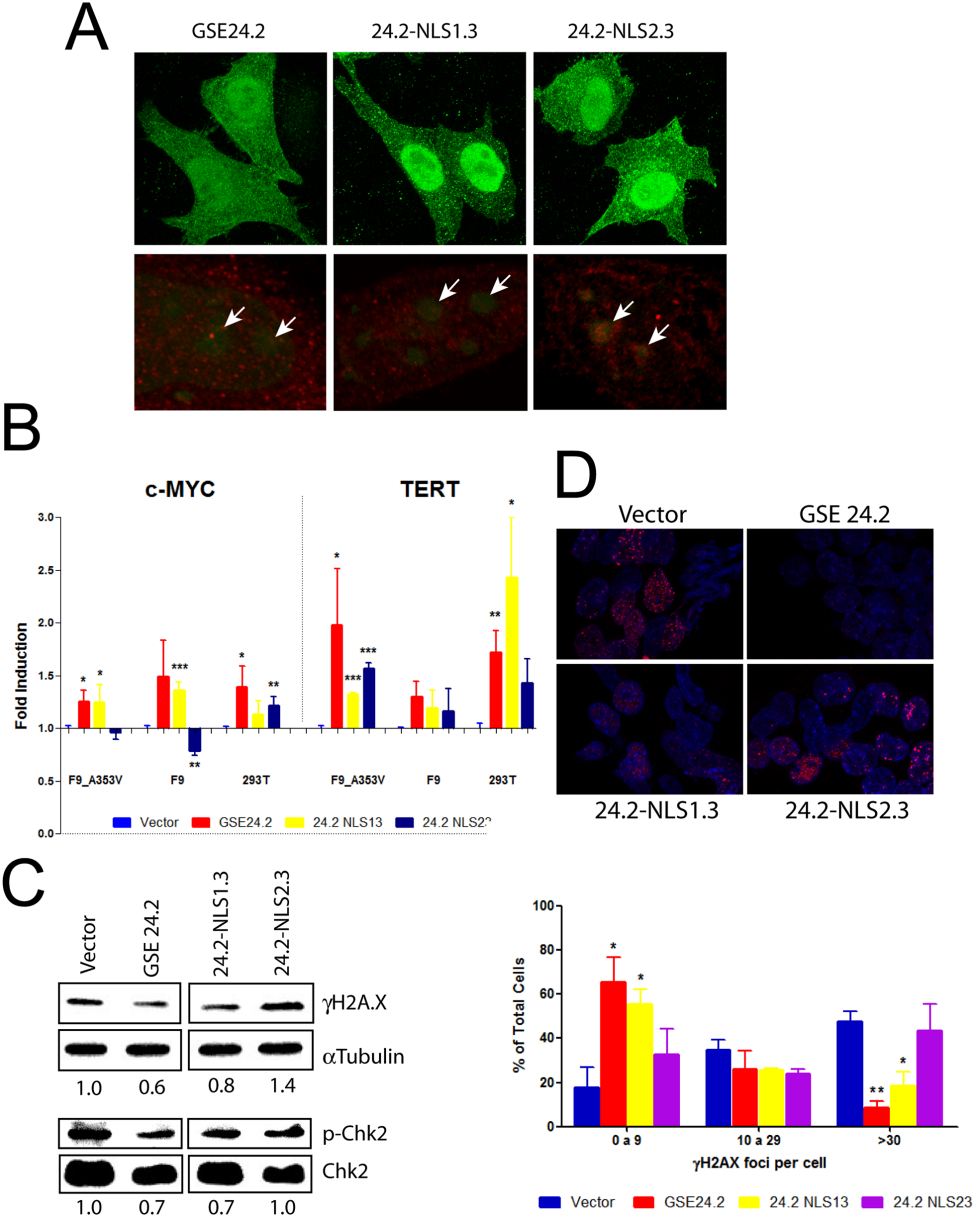
Functional analyses of GSE24.2-derived peptides containing nuclear localization signals. Panel A, cellular localization of the peptides. HeLa cells were transfected with pcDNA3 vectors expressing GSE24.2, GSE24.2-NLS1.3 and GSE24.2-NLS2.3 peptides fused to a N-terminal 6xmyc epitope, fixed, permeabilized and incubated with a c-myc antibody. Immunofluorescence staining of c-myc (green in top and red in the lower panels) was observed. Additionally, nucleoli were stained using 500 nM of the SYTO RNA Select Green (green in lower panels). Arrows indicate the position of nucleoli. Panel B, effect of pcDNA3_GSE24.2, GSE24.2_NLS13 and GSE24.2_NLS23 on the activity of c-myc and TERT promoters in F9_A353V, F9 and 293T cells. These three cell lines were cotransfected with pcDNA3 empty vector (Vector), GSE24.2 and GSE24.2-derived constructions (10 μg DNA/10^6^ cells) and c-myc-luc- or hTERT-luc -reporter vectors (1 μg/10^6^ cells). Luciferase activity was measured following 24 hours of transfection. Data were normalized by protein concentration and referred to the empty vector. Data points represent the mean and standard deviations of 3 experiments performed in triplicate (statistical significance: * p<0.05, **p<0.01, ***p<0.001). Panels C, D, analyses of cellular DNA damage. (C) F9_A353V cells were transfected with the pRRL-CMV-IRES-EGFP empty vector (Vector), or expressing GSE24.2, GSE24.2-NLS13 and GSE24.2-NLS23 (10 μg DNA/10^6^ cells). After 24 hours of transfection cells were lysed and the amount of γH2AX, α-tubulin (loading control), phosphorylated Chk2 (p-Chk2) and total Chk2 (Chk2) analyzed by western blot. The relative amounts of γH2AX/α-tubulin and p-Chk2/Chk2, normalized to the relation obtained for cells transfected with the empty vector, are indicated under each blot. (D) Upper panel. F9_A353V cells were transfected with the vectors indicated above and the presence of γH2AX analyzed by Immunofluorescence (red). Nuclear DNA was counterstained with DAPI (blue). Lower panel. Percentage of nuclei containing 0–9, 10–29 or more than 30 foci of γH2AX. More than 200 cells were analyzed for each transfection. Experiments were repeated 3 times with similar results. Asterisks indicate the statistical significance in relation to cells transfected with the empty vector (vector).

Dyskerin is a nucleolar protein and different cellular localization regions have been described [28]. Two of them were analyzed because their deletion or mutation altered the subcellular localization of dyskerin [28]. The first one (KRKR, named NLS1 in this article) is required for nuclear import while the second one (KKEKKKSKK, NLS2) increases the rate of nuclear and nucleolar import. Since GSE24.2 does not contain any of these regions, both were independently incorporated to the C-terminal region of the peptide to determine their possible contribution to the cellular localization and activity of the peptide. We used plasmid expression vectors with an N-terminal 6xmyc epitope (pcDNA3-myc). Cellular localization was studied by immunohystochemistry using α-myc antibodies after their expression in HeLa cells (Fig 3A). In some of these experiments nucleoli were identified using the specific SYTO RNA Select probe (Fig 3A, lower panels). The results showed that NLS1 incorporation increased the nuclear localization of GSE24.2, with low expression in nucleoli. In contrast, NLS2 incorporation resulted in an even expression in all nuclear regions. NLS1 and NLS2 incorporation to the N terminal region of GSE24.2 did not change the cellular localization (S1 Fig).

The functionality of the fusion peptides was determined by expressing them from the pcDNA3-myc vector in F9-A353V, F9 and HEK293T cells. We have previously described that expression of GSE24.2 activated TERT and c-myc promoters [18] [29]. Therefore, we studied the activity of the different constructions on the human c-myc and TERT promoters (Fig 3B). Both GSE24.2 and GSE24.2-NLS1.3 activated c-myc and TERT promoters in the three cell lines analyzed to similar levels although some of the activity increases were not statistically significant in F9 cells. However, GSE24.2-NLS2.3 only activated the TERT promoter. The effects of GSE24.2-NLS2.3 on the c-myc promoter were variable, a significant increase was observed in HEK293T cells and a significant decrease in F9 cells. None of the localization signals improved the transcriptional activity of GSE24.2. The possible activity on DNA damage was also studied using F9-A353V cells. The amount of DNA damage was estimated by evaluating the expression levels of phosphorylated **γ**H2AX and Chk2Thr68 proteins. Quantification of DNA damage was made by Western blot using anti **γ**H2AX and p-Chk2Thr68 antibodies (Fig 3C). In addition, the localization of DNA damage foci was observed by immunohistochemistry, evaluating the amount of **γ**H2AX-associated foci/cell (Fig 3D). GSE24.2 expression rescued the cells from DNA damage decreasing 40 and 30% respectively of **γ**H2AX and p-Chk2 levels (Fig 3C), as previously described [20]. NLS1.3 incorporation did not alter GSE24.2 activity while addition of NLS2.3 partially impaired this activity (Fig 3C and 3D).

The nuclear localization signal NLS1 was also added to the C-terminus of GSE4 to determine their possible influence on the activity of the peptide. GSE4 is similar to the conserved pseudouridine synthase active centre of other enzymes, where the Aspartic acid homologous to the one present at position 6 in GSE4 is always conserved [30]. Therefore, this Aspartic acid was changed to Alanine to test its possible functional relevance. These different peptides were either chemically synthesized or expressed in the cells from plasmid or lentiviral vectors, as indicated in each specific experiment.

**Fig 4:**
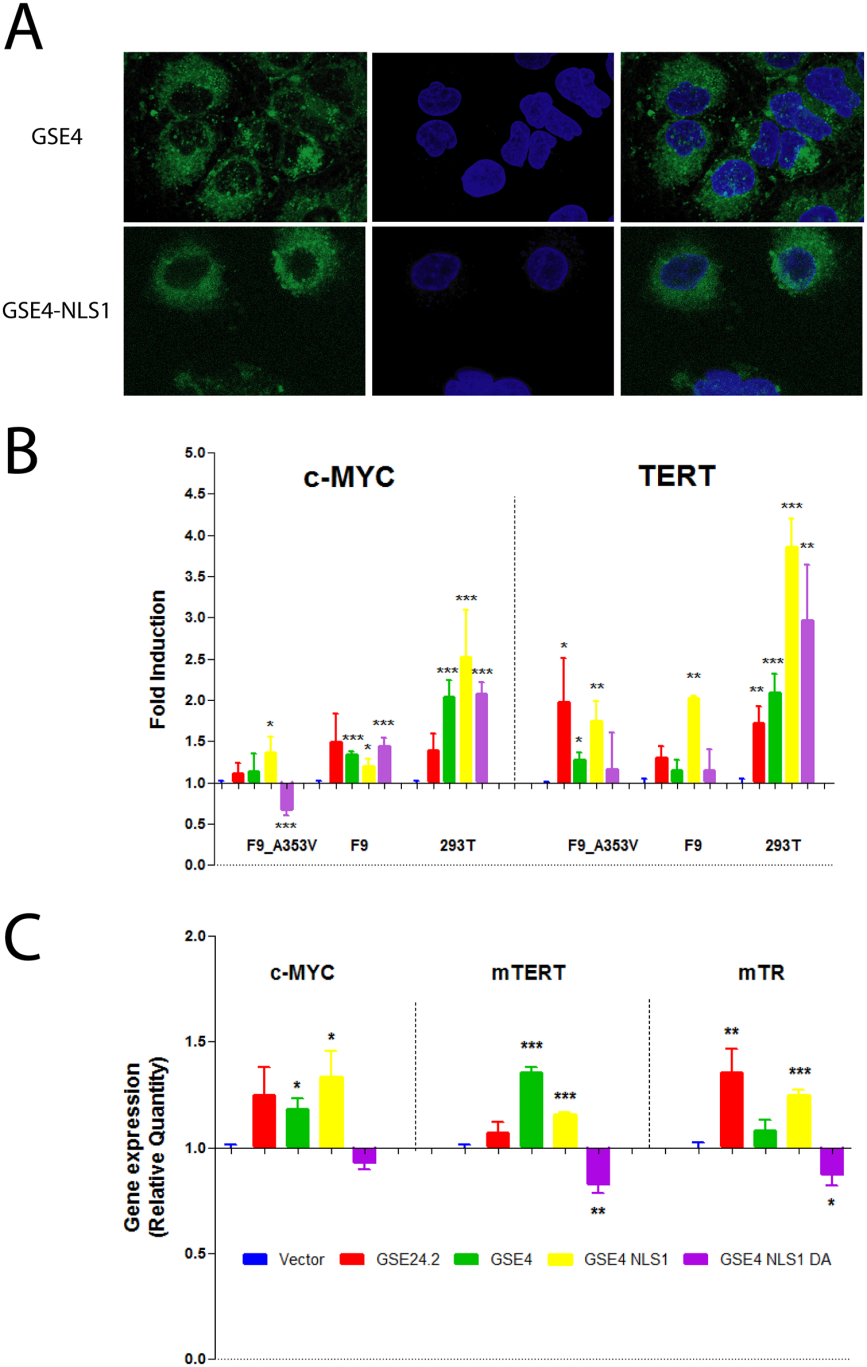
Cellular location and effect of GSE4 and derived peptides on promoter activity and gene expression. Panel A. HeLa cells were transfected with 15 μg of fluorescein-labelled GSE4 and GSE4_NLS1 peptides (green), fixed and visualized by confocal microscopy (left panels). DNA was counterstained with DAPI (blue, middle panels). Merged images are shown at the right panels. Panel B, promoter activity. F9-A353V, F9 and 293T cells were co-transfected with the pRRL-CMV-IRES-EGFP vector, either empty (Vector) or expressing the GSE24.2, GSE4, GSE4-NLSI or GSE4-NLS1-DA peptides (10 μg DNA/10^6^ cells) and c-myc-luc reporter or TERT-luc reporter vectors (1 μg/10^6^ cells). Luciferase activity was measured 24 hours after transfection. Data were normalized by protein concentration and referred to the activity of the control vector. Data points represent the mean and standard deviations of 3 experiments performed in triplicate. Panel C, F9-A353V cells were transfected with vectors indicated on panel B. Total RNA was extracted 24 h later and the levels of c-myc, TERT and TR(TERC) expression determined by reverse transcription and quantitative PCR. Expression levels were normalized by β-actin expression and referred to the expression levels of the cells transfected with the empty vector (Vector). The data shown represent the mean and standard deviations of 3 experiments performed in triplicate. Statistical significance: * p<0.05, **p<0.01, ***p<0.001.

The cellular localization of the GSE4 and GSE4-NLS1 peptides was studied by transfecting fluorescein-labelled peptides into HeLa cells. Both peptides showed a predominant cytoplasmic localization, as shown in Fig 4A, although the incorporation of NLS1 increased the presence of the peptide in the perinuclear region. The biological activity of the peptides was determined by expressing them from the lentiviral vector pRRL-CMV-IRES-GFP in different cell lines. Transcriptional activity was determined by using the previously mentioned reporter vectors containing the human c-myc and TERT promoters. The results obtained in F9-A353V, F9 and HEK293T cells are shown in Fig 4B. GSE24.2 activity was also determined to further compare the activity of the diverse peptides. GSE4 and GSE4-NLS1 peptides showed similar capacity than GSE24.2 to activate c-myc and TERT promoters in the cell lines analyzed, including the F9-A353V mutant cells. In HEK293T cells GSE4 and GSE4-NLS1 peptides were more active than GSE24.2 but that was not the case in F9 and F9-A353V cells. The mutated GSE4-NLS1-DA peptide was also active in these assays, with the exception of the c-myc promoter in F9-A353V cells where the activity was actually decreased, indicating that this Aspartic acid residue is not required for GSE4 activity. The activity of GSE4-NLS1-DA was, however, lower than that of GSE24.2, GSE4 and GSE4-NLS1 in F9-A353V cells. The addition of the nuclear localization signal NLS1 to GSE4 resulted in increased TERT promoter activity in the three cell lines analyzed (Fig 4B).

The expression of c-myc and TERT mRNAs, and TERC (TR) RNA, in transfected F9-A353V cells was determined by Reverse Transcription and quantitative PCR (Fig 4C). The results obtained are in general agreement with the reporter expression analyses (Fig 4B). GSE24.2, GSE4 and GSE4-NLS1 induced c-myc, TERT and TERC (TR) expression while GSE4-NLS1-DA repressed the expression of these RNAs in F9-A353V cells. GSE4 was more efficient in inducing TERT and less efficient in inducing TERC (TR) than GSE24.2 and GSE4-NLS1 in these experiments.

**Fig 5:**
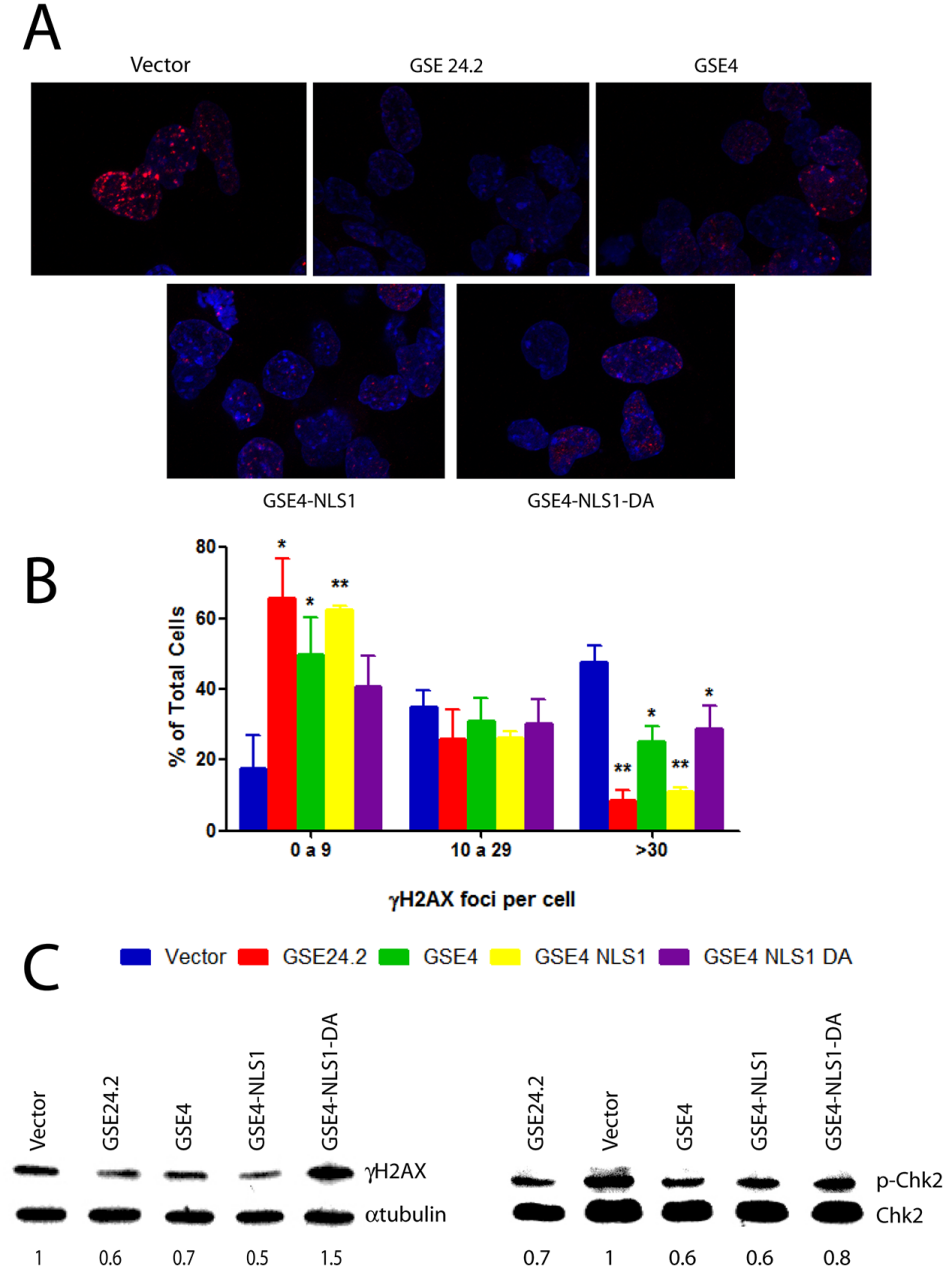
DNA damage protection by the expression of GSE4 and derived peptides. Panel A, F9-A353V cells were transfected with the pRRL-CMV-IRES-EGFP vector, either empty (vector), or expressing GSE24.2, GSE4, GSE4-NLS1 or GSE4_NLS1-DA (10 μg DNA/10^6^ cells). After 24 hours of transfection cells were fixed and incubated with anti γH2AX and a red-labeled secondary antibody. Nuclear DNA was counterstained with DAPI (blue). Panel B, the amount of γH2AX-expressing foci shown on panel A was determined. The percentage of cells containing 0 to 9, 10 to 29 or more than 30 foci is indicated. More than 200 cells were analyzed in each cell line. Experiments were repeated 3 times with similar results. Panel C, F9_A353V cells were transfected with the plasmids indicated on panel A. Twenty four hours after transfection cells were lysed and the expression of γH2AX, α-tubulin (loading control)(left panel) phosphorylated Chk2 (p-Chk2) and total Chk2 (Chk2)(right panel) analyzed by western blot. Numbers under each blot indicate the relative ɣH2AX/α-tubulin and p-Chk2/Chk2 expression normalized in relation to cells transfected with the empty vector (statistical significance: * p<0.05, **p<0.01, ***p<0.001).

The possible effect of the expression of GSE4 and derived peptides on DNA damage was also studied. F9-A353V cells were transfected with the expression vectors mentioned above. The amount of DNA damage in transfected cells was determined by quantification of **γ**H2AX-associated foci/cell (Fig 5A and 5B). In addition, Western blot analyses allowed the quantification of **γ**H2AX and p-Chk2Thr68 expression (Fig 5C). GSE4 and GSE4-NLS1 showed a protective effect on DNA damage similar to that of GSE24.2 in both assays. The mutant GSE4-NLS1-DA did not protect F9 A353V cells from DNA damage to the same extend under these experimental conditions. Altogether our results indicated that the functional activity of GSE4 peptide was similar to that GSE24.2 in all the biological assays performed in dyskeratosis congenita cells. Besides the addition of nuclear locations signals did not significantly increase the transcriptional activity of these peptides or their protection to DNA damage. In addition, the GSE4 Aspartic acid residue conserved in the pseudouridine synthase active centre of other enzymes, although not essential, is required for maximal activity of the peptide on dyskerin-mutant cells.

## Discussion

Telomere biology disorders, including dyskeratosis congenita, are characterized by the progressive shortening of the telomeres and by a number of cellular responses. In particular, telomere shortening and/or low telomerase activity induces in the cells a DNA damage response [31], and oxidative stress [32]. Therefore, the p53-dependent DNA damage response pathway is activated resulting in cell cycle arrest and cellular senescence [33, 34]. These responses have an important contribution to the pathology of the diseases through the deprivation of stem cells in the more proliferative tissues. We describe in this article that a dyskerin-derived peptide of eleven amino acids (GSE4) increases telomerase activity and decreases oxidative stress and the DNA damage response in cellular models of dyskeratosis congenita. Expression of GSE4 also regulates the global response of dyskeratosis congenita cells, increasing cell proliferation and reducing cellular senescence. A fibroblastoid cell line derived from a patient carrying the Ala2Val mutation in dkc1(patient 26 in ref [26]) was used for these studies.

The results described in the present article confirm those previously shown for the 55 amino acids long fragment of dyskerin, GSE24.2, that contains GSE4. GSE24.2 increases telomerase activity and decreases oxidative stress and the DNA damage response in dyskeratosis congenita patient cells [18, 19]. In addition, GSE4 protects F9-A353V dyskerin-mutant mouse cells from DNA damage and reduces oxidative stress, also as GSE24.2 [20]. Furthermore, we show in this article that GSE24.2 also increases cell proliferation and decreases cell senescence, which had not been reported previously. Therefore, we show that GSE4 presents the same biological activity than the longer peptide GSE24.2, which has been approved as an orphan drug for the treatment of dyskeratosis congenita. However, GSE4 presents some advantages over GSE24.2, derived from its smaller size. One of them is the lower cost of chemical synthesis. In addition, it could be easier to deliver into the cells. It would be also easier to develop variants that could have improved pharmaceutical characteristics.

The data presented also provide information about the mechanisms of action of these peptides. GSE4 increases telomerase activity in DKC1-mutated F9-A353V cells, increases expression of c-myc and the TERT and TERC telomerase components and the activity of TERT and c-myc promoters, as previously reported for GSE24.2 [19]. The activity of the TERT promoter has been shown to be higher in cancer cell lines than in transformed cells lines similar to those used in the present study [35] therefore, the possible effects of GSE4 on TERT expression in tumour cells should be tested in future studies. The structural studies indicate that GSE24.2 and GSE4 do not require nuclear localization signals for activity. The incorporation of nuclear or nucleolar localization signals to GSE24.2 increase nuclear or nucleolar localization but the transcriptional activity of the peptide and the protective role on DNA damage are not increased. Actually, the incorporation of the nucleolar localization signal NLS2 decreases GSE24.2 activity. The GSE4 peptide containing a nuclear localization signals (GSE4-NLS1) also show a biological activity similar to that of GSE4. These observations do not discard a nuclear function for GSE24.2 or GSE4 since the localization experiments indicate that GSE24.2 and GSE4, at a lower extend, are present both at the nucleus and the cytoplasm in the absence of nuclear localization signals, probably due to their small size.

Expression of GSE24.2 and GSE4 produces biological effects on several cell types expressing either Wild type or mutated dyskerin indicating that these peptides do not just complement dyskerin mutations. They seem to play several roles in the cell. It would be interesting to determine if there is a common mechanism for these activities or not. The data reported in this article indicate that some peptide variants differ in their activities depending on the assay and/or cell type studied. For example, GSE24.2-NLS2.3 activates TERT promoter at the same extend as GSE24.2 and GSE24.2-NLS1.3 but is less effective on c-myc promoter activation and on the protection of F9 A353V basal DNA damage. In the same sense, the GSE4-NLS1-DA variant increases transcriptional activity in F9 and HEK293T cells but is not active in the dyskerin-mutated F9-A353V cells neither in transcription activation nor in basal DNA damage protection. These data could indicate that these peptides participate in different cellular processes through different mechanisms. Some of the variant studied, like GSE4-NLS1-DA could require the presence of Wild-type dyskerin for some of their activities but other variants and the unmodified GSE24.2 and GSE4 peptides do not seem to be dependent on dyskerin for any of their biological activities.

In summary, the eleven amino acids long GSE4 peptide shows a protective effect on dyskerin mutated cells similar to the one previously shown for GSE24.2. Protection is mediated by increased telomerase activity and cell proliferation but also by decreased oxidative stress, DNA damage and cell senescence. These properties make of GSE4 a good candidate as a drug for dyskeratosis congenita treatment with some practical advantages over the already approved GSE24.2 peptide.

## Supporting Information

S1 Fig Cellular localization of the GSE24.2 peptide containing nuclear localization signals at the N-terminal region. pcDNA3 vectors were generated coding for the GSE24.2 peptide fused to the NLS1 nuclear localization signal (KRKR) or the NLS2 signal (KKEKKKSKK) fused to the N-terminal region, named 24.2-NLS1-5 and 24.2-NLS2.5 respectively. The nuclear localization signals were placed between the N-terminal 6xmyc epitope and the GSE24.2 peptide in the proteins expressed from these vectors. HeLa cells were transformed with the pcDNA3 vectors expressing GSE24.2, 24.2-NLS1.5 and 24.2-NLS2.5, fixed, permeabilized and incubated with a c-myc antibody. Immunofluorescence staining was observed after incubation of the preparations with a secondary antibody conjugated with Alexa fluor 488 using a Nikon 90i microscope.
(PDF)

## References

[1] Savage SA. Human telomeres and telomere biology disorders. Prog Mol Biol Transl Sci. 2014;125:41–66. Epub 2014/07/06. B978-0-12-397898-1.00002–5 [pii]. 10.1016/B978-0-12-397898-1.00002-5 . 24993697

[2] Meyne J, Ratliff RL, Moyzis RK. Conservation of the human telomere sequence (TTAGGG)n among vertebrates. Proc Natl Acad Sci U S A. 1989;86(18):7049–53. Epub 1989/09/01. . 2780561 10.1073/pnas.86.18.7049 PMC297991

[3] de Lange T, Shiue L, Myers RM, Cox DR, Naylor SL, Killery AM, et al Structure and variability of human chromosome ends. Mol Cell Biol. 1990;10(2):518–27. Epub 1990/02/01. . 2300052 10.1128/mcb.10.2.518-527.1990 PMC360828

[4] Greider CW, Blackburn EH. Identification of a specific telomere terminal transferase activity in Tetrahymena extracts. Cell. 1985;43(2 Pt 1):405–13. Epub 1985/12/01. 0092-8674(85)90170-9 [pii]. . 3907856 10.1016/0092-8674(85)90170-9

[5] Blackburn EH. Telomeres and telomerase: their mechanisms of action and the effects of altering their functions. FEBS Lett. 2005;579(4):859–62. Epub 2005/02/01. S0014-5793(04)01426-7 [pii]. 10.1016/j.febslet.2004.11.036 . 15680963

[6] Cohen SB, Graham ME, Lovrecz GO, Bache N, Robinson PJ, Reddel RR. Protein composition of catalytically active human telomerase from immortal cells. Science. 2007;315(5820):1850–3. Epub 2007/03/31. 315/5820/1850 [pii]. 10.1126/science.1138596 . 17395830

[7] Angrisani A, Vicidomini R, Turano M, Furia M. Human dyskerin: beyond telomeres. Biol Chem. 2014;395(6):593–610. Epub 2014/01/29. 10.1515/hsz-2013-0287 /j/bchm.just-accepted/hsz-2013-0287/hsz-2013-0287.xml [pii]. . 24468621

[8] Kiss T, Fayet E, Jady BE, Richard P, Weber M. Biogenesis and intranuclear trafficking of human box C/D and H/ACA RNPs. Cold Spring Harb Symp Quant Biol. 2006;71:407–17. Epub 2007/03/27. 10.1101/sqb.2006.71.025 . 17381323

[9] Wright WE, Tesmer VM, Huffman KE, Levene SD, Shay JW. Normal human chromosomes have long G-rich telomeric overhangs at one end. Genes Dev. 1997;11(21):2801–9. Epub 1997/11/14. . 9353250 10.1101/gad.11.21.2801 PMC316649

[10] Palm W, de Lange T. How shelterin protects mammalian telomeres. Annu Rev Genet. 2008;42:301–34. Epub 2008/08/06. 10.1146/annurev.genet.41.110306.130350 . 18680434

[11] Zou Y, Sfeir A, Gryaznov SM, Shay JW, Wright WE. Does a sentinel or a subset of short telomeres determine replicative senescence? Mol Biol Cell. 2004;15(8):3709–18. Epub 2004/06/08. 10.1091/mbc.E04-03-0207 E04-03-0207 [pii]. . 15181152 PMC491830

[12] Glousker G, Touzot F, Revy P, Tzfati Y, Savage SA. Unraveling the pathogenesis of Hoyeraal-Hreidarsson syndrome, a complex telomere biology disorder. Br J Haematol. 2015 Epub 2015/05/06. . 25940403 10.1111/bjh.13442 PMC4526362

[13] Dokal I. Dyskeratosis congenita in all its forms. Br J Haematol. 2000;110(4):768–79. Epub 2000/10/29. bjh2109 [pii]. . 11054058 10.1046/j.1365-2141.2000.02109.x

[14] Ge J, Yu YT. RNA pseudouridylation: new insights into an old modification. Trends Biochem Sci. 2013;38(4):210–8. Epub 2013/02/09. S0968-0004(13)00003-0 [pii]. 10.1016/j.tibs.2013.01.002 . 23391857 PMC3608706

[15] Schwartz S, Bernstein DA, Mumbach MR, Jovanovic M, Herbst RH, Leon-Ricardo BX, et al Transcriptome-wide Mapping Reveals Widespread Dynamic-Regulated Pseudouridylation of ncRNA and mRNA. Cell. 2014;159(1):148–62. Epub 2014/09/16. S0092-8674(14)01098-8 [pii]. 10.1016/j.cell.2014.08.028 . 25219674 PMC4180118

[16] Carlile TM, Rojas-Duran MF, Zinshteyn B, Shin H, Bartoli KM, Gilbert WV. Pseudouridine profiling reveals regulated mRNA pseudouridylation in yeast and human cells. Nature. 2014 Epub 2014/09/06. nature13802 [pii]. 10.1038/nature13802 . 25192136 PMC4224642

[17] Mason PJ, Bessler M. The genetics of dyskeratosis congenita. Cancer Genet. 2011;204(12):635–45. Epub 2012/01/31. S2210-7762(11)00308-5 [pii]. 10.1016/j.cancergen.2011.11.002 . 22285015 PMC3269008

[18] Machado-Pinilla R, Sanchez-Perez I, Murguia JR, Sastre L, Perona R. A dyskerin motif reactivates telomerase activity in X-linked dyskeratosis congenita and in telomerase-deficient human cells. Blood. 2008;111(5):2606–14. Epub 2007/12/07. blood-2007-04-083261 [pii]. 10.1182/blood-2007-04-083261 . 18057229

[19] Machado-Pinilla R, Carrillo J, Manguan-Garcia C, Sastre L, Mentzer A, Gu BW, et al Defects in mTR stability and telomerase activity produced by the Dkc1 A353V mutation in dyskeratosis congenita are rescued by a peptide from the dyskerin TruB domain. Clin Transl Oncol. 2012;14(10):755–63. Epub 2012/08/03. 10.1007/s12094-012-0865-4 . 22855157 PMC3643512

[20] Manguan-Garcia C, Pintado-Berninches L, Carrillo J, Machado-Pinilla R, Sastre L, Perez-Quilis C, et al Expression of the Genetic Suppressor Element 24.2 (GSE24.2) Decreases DNA Damage and Oxidative Stress in X-Linked Dyskeratosis Congenita Cells. PLoS One. 2014;9(7):e101424 Epub 2014/07/06. 10.1371/journal.pone.0101424 PONE-D-14-06623 [pii]. . 24987982 PMC4079255

[21] Mochizuki Y, He J, Kulkarni S, Bessler M, Mason PJ. Mouse dyskerin mutations affect accumulation of telomerase RNA and small nucleolar RNA, telomerase activity, and ribosomal RNA processing. Proc Natl Acad Sci U S A. 2004;101(29):10756–61. Epub 2004/07/09. 10.1073/pnas.0402560101 0402560101 [pii]. . 15240872 PMC490007

[22] Oh S, Song YH, Kim UJ, Yim J, Kim TK. In vivo and in vitro analyses of Myc for differential promoter activities of the human telomerase (hTERT) gene in normal and tumor cells. Biochem Biophys Res Commun. 1999;263(2):361–5. Epub 1999/09/24. 10.1006/bbrc.1999.1366 S0006-291X(99)91366-9 [pii]. . 10491298

[23] Wright WE, Shay JW, Piatyszek MA. Modifications of a telomeric repeat amplification protocol (TRAP) result in increased reliability, linearity and sensitivity. Nucleic Acids Res. 1995;23(18):3794–5. Epub 1995/09/25. l50206 [pii]. . 7479015 10.1093/nar/23.18.3794 PMC307284

[24] Sanchez-Perez I, Murguia JR, Perona R. Cisplatin induces a persistent activation of JNK that is related to cell death. Oncogene. 1998;16(4):533–40. Epub 1998/03/04. 10.1038/sj.onc.1201578 . 9484843

[25] Giulietti A, Overbergh L, Valckx D, Decallonne B, Bouillon R, Mathieu C. An overview of real-time quantitative PCR: applications to quantify cytokine gene expression. Methods. 2001;25(4):386–401. Epub 2002/02/16. 10.1006/meth.2001.1261 S1046-2023(01)91261-7 [pii]. . 11846608

[26] Carrillo J, Martinez P, Solera J, Moratilla C, Gonzalez A, Manguan-Garcia C, et al High resolution melting analysis for the identification of novel mutations in DKC1 and TERT genes in patients with dyskeratosis congenita. Blood Cells Mol Dis. 2012;49(3–4):140–6. Epub 2012/06/06. S1079-9796(12)00119-2 [pii]. 10.1016/j.bcmd.2012.05.008 . 22664374

[27] Blasco MA, Lee HW, Hande MP, Samper E, Lansdorp PM, DePinho RA, et al Telomere shortening and tumor formation by mouse cells lacking telomerase RNA. Cell. 1997;91(1):25–34. Epub 1997/10/23. S0092-8674(01)80006-4 [pii]. . 9335332 10.1016/s0092-8674(01)80006-4

[28] Heiss NS, Girod A, Salowsky R, Wiemann S, Pepperkok R, Poustka A. Dyskerin localizes to the nucleolus and its mislocalization is unlikely to play a role in the pathogenesis of dyskeratosis congenita. Hum Mol Genet. 1999;8(13):2515–24. Epub 1999/11/11. ddc279 [pii]. . 10556300 10.1093/hmg/8.13.2515

[29] Carrillo J, Gonzalez A, Manguan-Garcia C, Pintado-Berninches L, Perona R. p53 pathway activation by telomere attrition in X-DC primary fibroblasts occurs in the absence of ribosome biogenesis failure and as a consequence of DNA damage. Clin Transl Oncol. 2013;16(6):529–38. Epub 2013/09/26. 10.1007/s12094-013-1112-3 . 24065372

[30] Hamma T, Ferre-D'Amare AR. Pseudouridine synthases. Chem Biol. 2006;13(11):1125–35. Epub 2006/11/23. S1074-5521(06)00342-5 [pii]. 10.1016/j.chembiol.2006.09.009 . 17113994

[31] Gu BW, Bessler M, Mason PJ. A pathogenic dyskerin mutation impairs proliferation and activates a DNA damage response independent of telomere length in mice. Proc Natl Acad Sci U S A. 2008;105(29):10173–8. Epub 2008/07/16. 0803559105 [pii]. 10.1073/pnas.0803559105 . 18626023 PMC2481326

[32] Gu BW, Fan JM, Bessler M, Mason PJ. Accelerated hematopoietic stem cell aging in a mouse model of dyskeratosis congenita responds to antioxidant treatment. Aging Cell. 2011;10(2):338–48. Epub 2011/01/19. 10.1111/j.1474-9726.2011.00674.x . 21241452 PMC3238467

[33] Cesare AJ, Karlseder J. A three-state model of telomere control over human proliferative boundaries. Curr Opin Cell Biol. 2012;24(6):731–8. Epub 2012/09/06. S0955-0674(12)00132-9 [pii]. 10.1016/j.ceb.2012.08.007 . 22947495 PMC3532573

[34] Alder JK, Barkauskas CE, Limjunyawong N, Stanley SE, Kembou F, Tuder RM, et al Telomere dysfunction causes alveolar stem cell failure. Proc Natl Acad Sci U S A. 2015;112(16):5099–104. Epub 2015/04/05. 1504780112 [pii]. 10.1073/pnas.1504780112 . 25840590 PMC4413294

[35] Si SY, Song SJ, Zhang JZ, Liu JL, Liang S, Feng K, et al Cloning of mouse telomerase reverse transcriptase gene promoter and identification of proximal core promoter sequences essential for the expression of transgenes in cancer cells. Oncol Rep. 2011;26(2):377–82. Epub 2011/05/14. 10.3892/or.2011.1303 . 21567104

