# Supplementary material for: GSE4, a Small Dyskerin- and GSE24.2-Related Peptide, Induces Telomerase Activity, Cell Proliferation and Reduces DNA Damage, Oxidative Stress and Cell Senescence in Dyskerin Mutant Cells
Source: PLoS One. 2015 Nov 16;10(11):e0142980. doi: 10.1371/journal.pone.0142980 (PMC4646510; doi:10.1371/journal.pone.0142980)
Supplement: S1 Fig — pcDNA3 vectors were generated coding for the GSE24.2 peptide fused to the NLS1 nuclear localization signal (KRKR) or the NLS2 signal (KKEKKKSKK) fused to the N-terminal region, named 24.2-NLS1-5 and 24.2-NLS2.5 respectively. The nuclear localization signals were placed between the N-terminal 6xmyc epitope and the GSE24.2 peptide in the proteins expressed from these vectors. HeLa cells were transformed with the pcDNA3 vectors expressing GSE24.2, 24.2-NLS1.5 and 24.2-NLS2.5, fixed, permeabilized and incubated with a c-myc antibody. Immunofluorescence staining was observed after incubation of the preparations with a secondary antibody conjugated with Alexa fluor 488 using a Nikon 90i microscope. (PDF) [file pone.0142980.s001.pdf]

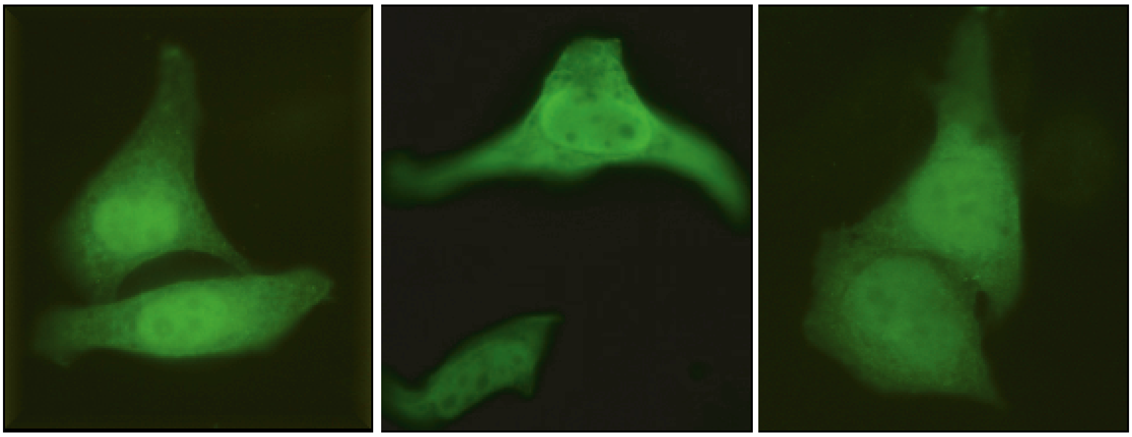

Figure S1. Cellular localization of the GSE24.2 peptide containing nuclear localization signals at the N-terminal region.

pcDNA3 vectors were generated coding for the GSE24.2 peptide fused to the NLS1 nuclear localization signal (KRKR) or the NLS2 signal (KKEKKKSKK) fused to the N-terminal region, named 24.2-NLS1-5 and 24.2-NLS2.5 respectively. The nuclear localization signals were placed between the N-terminal 6xmyc epitope and the GSE24.2 peptide in the proteins expressed from these vectors. HeLa cells were transformed with the pcDNA3 vectors expressing GSE24.2, 24.2-NLS1.5 and 24.2-NLS2.5, fixed, permeabilized and incubated with a c-myc antibody. Immunofluorescence staining was observed after incubation of the preparations with a secondary antibody conjugated with Alexa fluor 488 using a Nikon 90i microscope.

Materials and Methods

The fragments were obtained by amplification from the pcDNA-GSE24.2 vector using the following oligonucleotides:

GSE24.2-NLS1.5 forward oligonucleotide: 5'-GGGAATTCTAAGCGGAAGCGAGG-TTTCATTAATCTTGACAAGC-3'.

GSE24.2-NLS2.5 forward oligonucleotide: 5'- GGGAATTCTAAGAAGGAAAAGAA-GAAGAGTAAGAAGGGTTTCATTAATCTTGACAAGC-3'

Common reverse oligonucleotide: 5'-GGTCTAGACTTCACCAAGCGAGTGGCTCG-3'.

Amplified DNA fragments were digested with EcoRI and XbaI and cloned into the pcDNA3-myc vector digested with the same restriction enzymes.
